# Impact of Adjunctive Therapy with *Chlorellav ulgaris* Extract on Antioxidant Status, Pulmonary Function, and Clinical Symptoms of Patients with Obstructive Pulmonary Diseases

**DOI:** 10.3797/scipharm.1202-06

**Published:** 2012-06-18

**Authors:** Yunes Panahi, Sasan Tavana, Amirhossein Sahebkar, Homeira Masoudi, Nima Madanchi

**Affiliations:** 1 Chemical Injuries Research Center, Baqiyatallah University of Medical Sciences, Tehran, Iran.; 2 Clinical Research and Development Center, Shahid Modarres Hospital, Shahid Beheshti University of Medical Sciences, Tehran, Iran.; 3 Biotechnology Research Center and School of Pharmacy, Mashhad University of Medical Sciences, Mashhad, Iran.; 4 Pharmaceutical Sciences Branch, Islamic Azad University, Tehran, Iran.; 5 Faculty of Medicine, Tehran University of Medical Sciences, Tehran, Iran.

**Keywords:** *Chlorella vulgaris*, Lung, Bronchospasm, Inflammation, Oxidative stress, Spirometry

## Abstract

This present trial investigated the efficacy of supplementation with *Chlorella vulgaris*, a bioactive microalga rich in macro- and micronutrients, in the improvement of biochemical and clinical symptoms in patients with obstructive pulmonary disorders. Ninety-seven patients with chronic obstructive pulmonary disease (COPD) or asthma who were under conventional treatment regimens were randomly assigned to *C. vulgaris* extract (CVE) (*n*=48; 2700 mg/day) or no adjunctive therapy (*n*=49) for eight weeks. Serum levels of antioxidants along with spirometric parameters and clinical symptoms were evaluated pre- and post-trial. The magnitude of increases in the concentrations of glutathione, vitamin E, and vitamin C, and activities of glutathione peroxidase, catalase, and superoxide dismutase enzymes were all significantly greater in the CVE vs. control group (*p*<0.05). In spite of increases, none of the assessed spirometric parameters (FVC, FEV1, FEV1/FVC, and FEF_25–75%_) did significantly differ by the end of the trial in the study groups, apart from a significant elevation of FEV1 in the control group (*p*=0.03). The frequency of coughing, shortness of breath, wheezing, and sputum brought up were all significantly reduced in both CVE and control groups (*p*<0.05). The rate of improvement for sputum brought up and wheezing were significantly greater in the CVE group compared to the control group (*p*<0.05). Although CVE was found to ameliorate serum antioxidant status, its supplementation was not associated with any bronchodilatory activity. The results of the present trial do not support any clinical efficacy for CVE in patients with obstructive pulmonary disorders.

## Introduction

Chronic obstructive pulmonary disease (COPD) is among the most prevalent disorders of the respiratory tract, ranked as the 5^th^ cause of mortality worldwide [1]. Asthma is also another prevalent chronic respiratory disorder which has many characteristics, in terms of symptoms and pathophysiologic features, in common with COPD. Regarding the unknown pathophysiologic nature of these disorders and, on the other hand, contribution of different predisposing factors (e.g. environmental and genetic factors), treatment of asthma and COPD has mainly relied on symptomatic management and increasing the quality of life [2].

Treatment of COPD and asthma is life-long and usually based on pharmacotherapy with bronchodilators such as B_2_ agonists and inhaled glucocorticoids. On the other hand, long-term use of such medications has been linked with several adverse effects. For instance, glucocorticoids may predispose patients to osteoporosis, hypertension, hyperglycemia, and cataracts. In the same manner, B_2_ agonists could induce side effects such as tremor, hypokalemia, and myasthenia [1]. Therefore, introduction of novel adjunctive therapies that could reduce the required dose of conventional therapies is of high priority and would lessen the incidence of adverse effects, while increasing the compliance of patients.

Among the many risk factors which contribute to the pathogenesis of COPD and asthma, the role of oxidative stress and inflammation appears to be more prominent [3, 4]. Besides, increasing evidence has been found on the involvement of autoimmunity mechanisms in the pathophysiology of these disorders [5]. Therefore, mitigation of oxidative stress and inflammation along with modulation of the immune system are among the main targets for successful management of asthma and COPD.

*Chlorella vulgaris,* a green, single-celled microalga, has been used as a food supplement and alternative medicine in Japan and other Asian countries for hundreds of years. What makes this microorganism unique is its well-balanced content of numerous macro- and micronutrients including carbohydrates, proteins, nucleic acids, essential amino acids, fatty acids (Ω-3 and Ω-6), vitamins, dietary fiber, growth factors, and so forth [6, 7]. In spite of the richness of functional nutrients in *C. vulgaris*, investigations regarding the biological activities of these microalgae have been relatively few. Hence, the present trial set out to explore the therapeutic efficacy of adjunctive therapy with *C. vulgaris* extract (CVE) in patients with asthma or COPD receiving conventional treatment regimens.

## Methods

This was a randomized open-label clinical trial, investigating the impact of adjunctive therapy with CVE on the improvement of spirometric parameters and oxidative stress biomarkers in patients with chronic pulmonary disease. Included subjects were patients with COPD (stage I or II) or asthma referring to the Baqiyatallah and Modarres Hospitals (Tehran, Iran). Exclusion criteria were hypersensitivity to algal preparations, pregnancy, presence of cardiovascular disease, age > 70 y, consumption of antioxidant drugs or supplements, noncooperation in performing spirometry, exacerbation of disease within the preceding three months, and not consuming the supplement after one week of the trial beginning.

Ninety-seven patients (mean age: 50.9 ± 14.9) were recruited. All patients were under standard anti-asthma/anti-COPD treatment regimens. Forty-eight of the recruited patients were randomly assigned to CVE as an adjunct to their standard respiratory therapy. The remaining patients (n = 49) were followed-up as the control group.

CVE was administered at a dose of 2700 mg/day (900 mg TID) for eight weeks. A complete explanation about the intervention and its effects was given to all participants. The study was approved by the institutional Ethics Committee, and written informed consent was obtained from all patients.

The CVE used in the study was in the form of 300 mg tablets which are commercially available under the trade name ALGOMED^®^ (Bioprodukte Prof. Steinberg Produktions- und Vertriebs GmbH & Co KG, Klötze, Germany). The tablets contained 98% *C. vulgaris* powder, 1% separating agent (silicic acid), and 1% plant-based magnesium stearate). The tablets were ~ 9 mm in diameter and ~ 300 mg in weight. The ingredients of the tablets are summarized in Table 1 (based on the manufacturer’s information).

Fasting serum samples were collected at the baseline as well as at the end of trial, and were analyzed for vitamin C, vitamin E, malonedialdehyde (MDA), and glutathione (GSH) concentrations, superoxide dismutase (SOD), glutathione peroxidase (GPx), and catalase (CAT) activities, and total antioxidant status (TAS). In addition, all participants underwent spirometry (HI-801 Chest M.I. Spirometer, Tokyo, Japan) at the baseline and at the end of the trial. The spirometer was calibrated using the device provided by the manufacturer. To assess pulmonary function, forced expiratory volume in the first second (FEV1), forced vital capacity (FVC), FEV1/FVC ratio, and forced expiratory flow 25–75% (FEF_25–75%_) were measured. The frequencies of coughing, shortness of breath, wheezing, and sputum brought up were also recorded for participants at the baseline and at the end of the trial. Assessment of the clinical symptoms was performed through interviews and based on the St. George’s respiratory questionnaire for COPD patients [8].

Statistical analyses were performed using SPSS software (version 17.0). Data were expressed as mean ± SEM. A per protocol statistical approach was used for data analysis. Between- and within-group comparisons were performed using independent and paired samples *t*-test, respectively. A two-sided *p*-value of < 0.05 was considered as statistically significant.

## Results

**Fig. 1:**
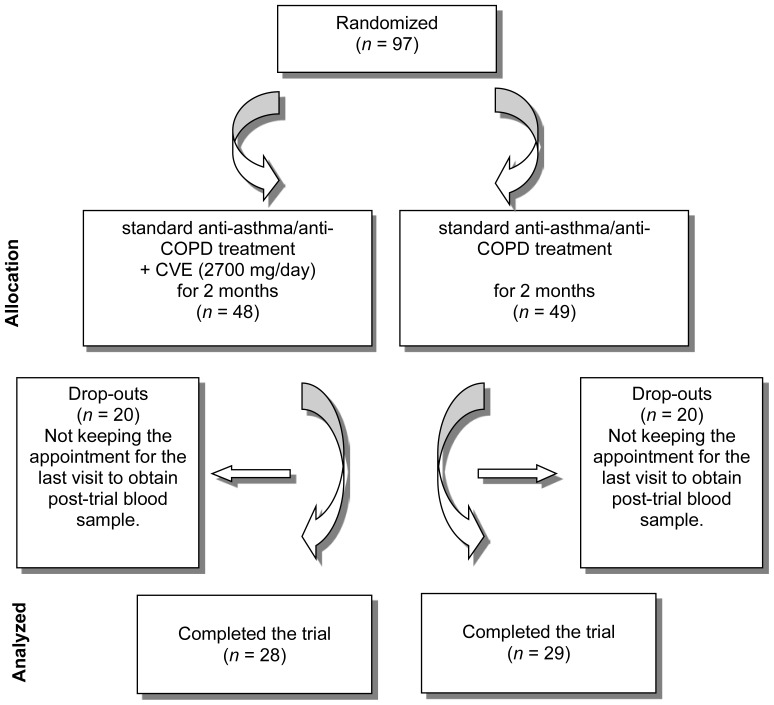
Flowchart of the trial.

Of the 97 recruited patients who initially entered into the trial, 57 completed the trial (Figure 1). Demographic characteristics of the study groups are summarized in Table 2.

### Effect of CVE supplementation on pulmonary function

**Fig. 2:**
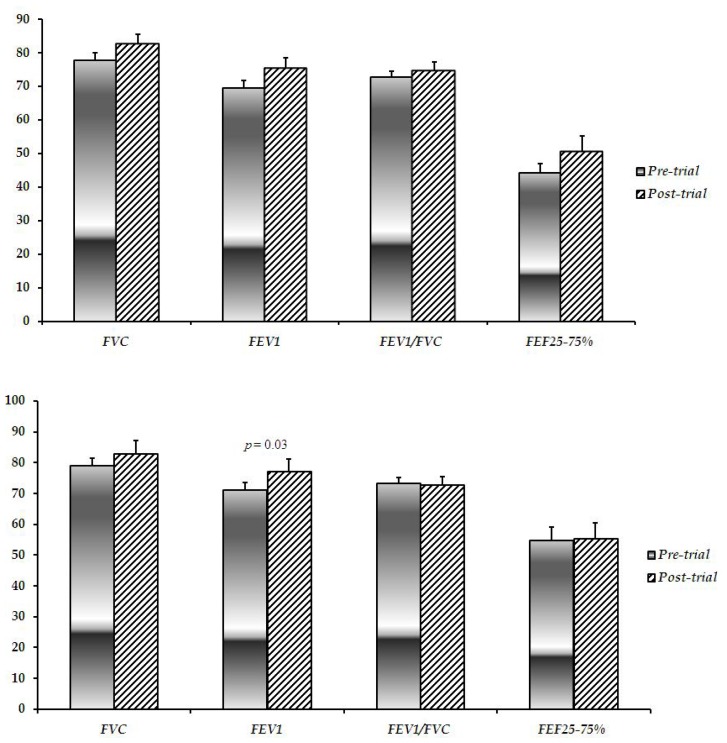
Baseline and post-trial pulmonary function test parameters in the CVE (up) and control (down) groups. CVE: *C. vulgaris* extract; FVC: forced vital capacity; FEV1: forced expiratory volume in the first second; FEF_25–75%_: forced expiratory flow 25–75%.

CVE and control groups were comparable regarding their baseline spirometric characteristics including FVC, FEV1, FEV1/FVC, and FEF_25–75%_ (*p* > 0.05). In the same manner, no significant difference was observed between the groups in the aforementioned parameters at the end of the trial (*p* > 0.05). In spite of increases, none of the assessed spirometric parameters did significantly differ by the end of the trial in the Chlorella group (*p* > 0.05; Figure 2). There was also a similar trend in the control group, apart from a significant elevation in the FEV1 (*p* = 0.03; Figure 2).

### Effect of CVE supplementation on serum oxidative stress biomarkers

No significant difference was observed between the groups regarding their baseline levels of oxidative stress biomarkers including MDA, vit E, vit C, GSH, SOD, GPx, CAT, TAS, and MDA (*p* > 0.05). However, baselines values for MDA (*p* < 0.001) and GPx (*p* = 0.009) were significantly higher in the CVE group compared to the control group. In the control group, serum GSH (*p* = 0.015) concentrations along with SOD (*p* < 0.001) activity and TAS (*p* < 0.001) were significantly increased by the end of trial, while CAT activity had significantly decreased (*p* = 0.05). No difference occurred for serum vit E, vit C, and GPx (*p* > 0.05). In contrast, all assessed antioxidant parameters had remarkably increased in the Chlorella group compared to their baseline levels (*p* < 0.001 for vit C, GSH, SOD, GPx, CAT, and TAS, and *p* = 0.002 for vit E). MDA levels were reduced by the end of the trial in both of the CVE (*p* < 0.001) and control (*p* = 0.016) groups (Table 3). The magnitude of changes in MDA (*p* < 0.001), GSH (*p* < 0.001), GPx (*p* < 0.001), CAT (*p* < 0.001), SOD (*p* = 0.011), vit C (*p* < 0.001), and vit E (*p* = 0.025) were all significantly greater in the CVE vs. the control group. The only exception was TAS, for which the magnitude of changes did not differ between the groups (*p* > 0.05) (Table 4).

### Effect of CVE on clinical symptoms of asthma and COPD

The effect of adjunctive therapy with CVE was evaluated on the severity of clinical symptoms of asthma and COPD. The severity and frequency of coughing, shortness of breath, wheezing, and sputum brought up were all significantly reduced in both of the CVE and control groups (*p* ≤ 0.001). The rate of improvement in the severity and frequency of sputum brought up and wheezing was significantly greater in the CVE group compared to the control group (*p* < 0.05). In contrast, the rate of improvement in coughing and sputum production was comparable between the groups (*p* > 0.05) (Table 5).

## Discussion

In spite of extensive research, the therapies that are currently available for the management of COPD and asthma are mainly symptomatic and associated with several adverse effects. These issues necessitate the use of alternative medicine in order to enhance the therapeutic efficacy of current medications. Although herbal remedies have long been used for the treatment of COPD, there has been relatively little focus in modern scientific research on the application of alternative/herbal remedies as adjunctive therapies [9]. The present study was designed to determine the effect of adjunctive therapy with CVE on spirometric function, oxidative stress biomarkers, and clinical symptoms of patients with asthma and COPD.

To date, several lines of evidence have supported the role of oxidative stress as a pathologic contributor to the inflammatory lung diseases including asthma and COPD [10, 11]. This oxidative burden could be secondary to the heightened state of inflammation which causes the influx and activation of macrophages, neutrophils, and eosinophils to the airways. These inflammatory cells are rich sources of ROS and exert direct oxidative damage characterized by increased lipid peroxidation, and vascular and tissue permeability [12, 13]. The expression, activity and/or concentration of different antioxidant measures such as SOD, GPx, CAT, vit E, and vit C have been reported to be decreased in patients with asthma or COPD. In addition, elevated levels of MDA, a widely-used biomarker of lipid peroxidation, has been reported in asthmatic and COPD patients [11]. Oxidant species impair mucocilliary clearance, and induce mucus hypersecretion and apoptosis in the alveolar epithelial cells and lung mesenchymal cells, all of which leading to airway damage that is the characteristic of COPD [11]. In light of the present findings, supplementation with CVE could effectively counterbalance all oxidative stress-induced alterations and thus prevent airway remodeling in asthmatic and COPD patients. In addition, antioxidant supplementation has been found to be beneficial in terms of improving pulmonary function, physical performance and exercise tolerance, and prevention of exacerbation episodes [14–16].

*Chlorella* has been known as a functional food and supernatural supplement since ancient times in countries like Japan and Taiwan. Interestingly, CVE contains a wide variety of different antioxidants including chlorophyll, α-carotene, β-carotene, ascorbic acid, α-tocopherol, lutein, lycopene, zeaxanthin, as well as trace elements such as zinc, copper, and magnesium which are crucial for the function of antioxidant metalloenzymes [6, 7] (Table 5). The antioxidant activities of *Chlorella* have been previously shown in both *in vitro* and different *in vivo* models of streptozocin-induced diabetes [17], atherosclerosis [18], hypertension [19], dementia [20], cadmium toxicity [21], and naphthalene toxicity [22].

The present study was limited in some ways. First, the high rate of dropouts caused the reduction of statistical power for the detection of significant differences between the groups. These dropouts were those who were lost to follow-up, mainly due to missing the appointment for the last visit to obtain a post-trial blood sample. However, it does not appear that the dropouts are related to any adverse effect of CVE, as the rate of drops is the same in both groups. Second, the duration of the study (two months) is relatively short for a definitive judgment on the benefits of CVE supplementation in patients with obstructive pulmonary disorders. Third, dietary intake of antioxidants during the course of the trial might have affected serum antioxidant status and hence, be a potential source of error.

To conclude, although CVE was found to ameliorate serum antioxidant status, its supplementation was not associated with any bronchodilatory activity. Hence, the results of the present trial (as the first ever to be performed for this purpose) do not support any clinical efficacy for CVE in patients with obstructive pulmonary disorders. In order to attain a more definitive judgment, future studies need to be focused on specific subcategories of chronic obstructive pulmonary disorders, and to assess the antioxidant status in respiratory samples (e.g. bronchoalveolar lavage fluid) as well. Finally, the impact of antioxidant therapy with CVE on spirometric parameters is suggested to be more closely evaluated in a long-term follow-up.

## Figures and Tables

**Tab. 1:** Ingredients of *C. vulgaris* extract tablets and their respective amounts.

Ingredient	Quantity
Fat (g/100g)	8.65
Protein (g/100g)	52.0
Carbohydrates (g/100g)	13.6
Ash (g/100g)	6.56
Water (g/100g)	3.63
Dietary fiber (g/100g)	15.6
Energy (Kcal/100g)	340

**Fatty acids**	

Saturated fatty acid (g/100g)	2.16
Monounsaturated fatty acid (g/100g)	1.69
Poly unsaturated fatty acid (g/100g)	3.34
Trans fatty acid (g/100g)	0.06

**ω-3 fatty acids**	

Linoleic acid (g/100g)	1.282
α-Linolenic acid (g/100g)	1.964

**ω-6 fatty acids**	

Octodecatetaenoic acid (g/100g)	0.003
Eicosadienoic acid (g/100g)	0.011
Arachidonc acid (g/100g)	0.009
Docosatetraenoic acid (g/100g)	0.020

**Miscellaneous**	

Lutein (mg/100g)	84.3
Lycopin (mg/100g)	0.307
Zeaxanthin (mg/100g)	0.679
Chlorophyll (g/kg)	15.21

**Vitamins**	

β-Carotene (mg/100g)	180.8
Vitamin B1 (mg/100g)	1.5
Vitamin B2 (mg/100g)	4.8
Vitamin B3 (mg/100g)	23.8
Vitamin B5 (mg/100g)	1.3
Vitamin B6 (mg/100g)	1.7
Vitamin B12 (μg/100g)	125.9
Vitamin C (mg/100g)	15.6
Folic acid (μg/100g)	26.9
Biotin (μg/100g)	191.6
Para-aminobenzoic acid (mg/100g)	0.6

**Minerals**	

Phosphorus (mg/100g)	959
Potassium (mg/kg)	21450
Magnesium (mg/kg)	4425
Calcium (mg/kg)	2710
Iron (mg/kg)	680
Copper (mg/kg)	19.0
Zinc (mg/kg)	54.5
Manganese (mg/kg)	39.5
Iodine (mg/kg)	12.9
Chromium (mg/kg)	0.575

Administered *C. vulgais* extract tablets were from Bioprodukte Prof. Steinberg (Produktions- und Vertriebs GmbH & Co KG, Klötze, Germany).

**Tab. 2:** Demographic characteristics of the study groups.

Parameter	CVE		Control	
	
	Male	Female	Male	Female
Gender	54.2%	45.8%	69.4%	30.6%
Age (y)	50.5 ± 3.2	48.1 ± 2.9	52.8 ± 2.8	51.6 ± 3.8
Height (cm)	173.7 ± 1.7	156.8 ± 1.2	169.1 ± 2.3	159.1 ± 2.2
Weight (kg)	78.4 ± 3.1	74.3 ± 2.3	75.5 ± 2.8	75.8 ± 4.3

**Past medical history**				

Cardiovascular disease	4.1%		4.1%	
Diabetes	2.0%		4.1%	
Hypertension	2.0%		8.1%	
Other	2.0%		2.0%	

**Tab. 3:** Pre- vs. post-trial comparison of oxidative stress biomarkers in the study groups.

	CVE			Control		
	
	Pre-trial	Post-trial	*p*-Value	Pre-trial	Post-trial	*p*-Value
**Vit E**	0.84 ± 0.12	3.62 ± 15.89	0.002	0.86 ± 0.11	0.90 ± 0.24	0.260
**Vit C**	0.82 ± 0.12	1.28 ± 0.33	<0.001	0.82 ± 0.13	0.77 ± 0.16	0.189
**GSH**	22.86 ± 1.15	32.23 ± 2.38	<0.001	22.69 ± 4.16	22.22 ± 4.61	0.015
**GPx**	4.63 ± 0.34	6.89 ± 0.53	<0.001	4.29 ± 0.69	4.44 ± 1.04	0.416
**CAT**	42.84 ± 3.21	53.35 ± 3.36	<0.001	41.72 ± 3.87	39.99 ± 4.92	0.050
**SOD**	2.33 ± 0.29	3.34 ± 0.48	<0.001	2.53 ± 0.48	3.13 ± 0.54	< 0.001
**TAS**	1.40 ± 0.19	1.69 ± 0.21	<0.001	1.44 ± 0.24	1.64 ± 0.27	< 0.001
**MDA**	9.76 ± 0.92	7.73 ± 0.71	<0.001	8.86 ± 0.77	8.26 ± 1.35	0.016

MDA: malonedialdehyde; vit E: vitamin E; vit C: vitamin C; GSH: glutathione; GP_X_: glutathione peroxidase; CAT: catalase; SOD: superoxide dismutase; TAS: total antioxidant status.

**Tab. 4:** Magnitude of changes in oxidative stress biomarkers in the study groups.

	CVE	Control	*p*-value
**MDA**	−2.03 ± 0.86	−0.60 ± 1.47	0.025
**VIT E**	2.78 ± 15.88	0.05 ± 0.25	<0.001
**VIT C**	0.46 ± 0.34	−0.04 ± 0.20	<0.001
**GSH**	9.37 ± 2.34	2.60 ± 5.20	<0.001
**GP_X_**	2.26 ± 0.44	0.15 ± 1.12	<0.001
**CAT**	10.51 ± 5.01	−1.73 ± 5.26	<0.001
**SOD**	1.01 ± 0.56	0.60 ± 0.78	0.011
**TAL**	0.30 ± 0.24	0.20 ± 0.29	0.104

MDA: malonedialdehyde; vit E: vitamin E; vit C: vitamin C; GSH: glutathione; GP_X_: glutathione peroxidase; CAT: catalase; SOD: superoxide dismutase; TAS: total antioxidant status.

**Tab. 5:** Comparison of clinical symptoms between the study groups.

	Frequency	CVE group			Control group			*p*-Value	
		
		Pre-trial	Post- trial	*p*- value	Pre-trial	Post- trial	*p*- value	pre- trial	post- trial
**Coughing**	Almost every day	47.8%	3.5%		59.2%	13.8%			
	A few days a week	21.8%	25.0%		10.2%	6.8%			
	A few days a month	6.5%	10.7%		6.1%	10.3%			
	Only with lung/respiratory infections			<0.001			<0.001	0.66	0.35
		21.8%	35.7%		20.4%	27.5%			
	Not at all	2.2%	25.0%		4.1%	41.3%			

**Shortness of breath**	Almost every day	73.9%	10.7%		67.3%	13.7%			
	A few days a week	17.3%	25.0%		20.4%	10.3%			
	A few days a month	2.2%	17.8%		6.1%	27.6%			
	Only with lung/respiratory infections			<0.001			<0.001	0.49	0.70
		4.3%	21.4%		6.1%	20.6%			
	Not at all	2.2%	25.0%		0.0%	27.6%			

**Wheezing**	Almost every day	84.7%	4.3%		63.2%	6.8%			
	A few days a week	8.6%	42.8%		18.3%	13.7%			
	A few days a month	4.3%	8.6%		4.1%	24.1%			
	Only with lung/respiratory infections			<0.001			<0.001	0.01	0.07
		2.1%	4.3%		8.1%	10.3%			
	Not at all	0.0	17.3		6.1	44.8			

**Sputum brought up**	Almost every day	47.8	3.5		59.2	24.1			
	A few days a week	21.8	7.1		10.2	17.24			
	A few days a month	6.5	10.7		6.1	6.8			
	Only with lung/respiratory infections			<0.001			<0.001	0.50	0.02
		21.8	57.1		20.4	37.9			
	Not at all	2.2	21.4		4.1	13.7			

## References

[1] Rabe KF, Hurd S, Anzueto A, Barnes PJ, Buist SA, Calverley P, Fukuchi Y, Jenkins C, Rodriguez-Roisin R, van Weel C, Zielinski J (2007). Global Initiative for Chronic Obstructive Lung Disease. Global strategy for the diagnosis, management and prevention of chronic obstructive pulmonary disease: GOLD executive summary. Am J Respir Crit Care Med.

[2] Siafakas NM, Vermeire P, Pride NB, Paoletti P, Gibson J, Howard P, Yernault JC, Decramer M, Higenbottam T, Postma DS, Rees J (1995). Optimal assessment and management of chronic obstructive pulmonary disease (COPD). Eur Respir J.

[3] Tkacova R, Kluchova Z, Joppa P, Petrasova D, Molcanyiova A (2007). Systemic inflammation and systemic oxidative stress in patients with acute exacerbations of COPD. Respir Med.

[4] MacNee W (2001). Oxidative stress and lung inflammation in airways disease. Eur J Pharmacol.

[5] Agustí A, MacNee W, Donaldson K, Cosio M (2003). Hypothesis: Does COPD have an autoimmune component?. Thorax.

[6] Borowitzka MA, Borowitzka LJ (1988). Vitamins and fine chemicals from micro-algae. Micro-algal biotechnology.

[7] Schubert LE, Round FE, Chapman DJ (1988). The use of spirulina and Chlorella as food resource for animals and humans. Progressing physiological research.

[8] Jones PW, Quirk FH, Baveystock CM (1991). The St George’s Respiratory Questionnaire. Respir Med.

[9] Guo R, Pittler MH, Ernst E (2006). Herbal medicines for the treatment of COPD: a systematic review. Eur Respir J.

[10] Kirkham P, Rahman I (2006). Oxidative stress in asthma and COPD: antioxidants as a therapeutic strategy. Pharmacol Ther.

[11] Lin JL, Thomas PS (2010). Current Perspectives of Oxidative Stress and its Measurement in Chronic Obstructive Pulmonary Disease. COPD.

[12] Dworski R (2000). Oxidant stress in asthma. Thorax.

[13] Harik-Khan RI, Muller DC, Wise RA (2004). Serum vitamin levels and the risk of asthma in children. Am J Epidemiol.

[14] Stav D, Raz M (2009). Effectof N-acetylcysteine on airtrapping in COPD: a randomized placebo-controlled study. Chest.

[15] Sutherland ER, Crapo JD, Bowler RP (2006). N-acetylcysteine and exacerbations of chronic obstructive pulmonary disease. COPD.

[16] Zuin R, Palamidese A, Negrin R, Catozzo L, Scarda A, Balbinot M (2005). High-dose N-acetylcysteine in patietns with exacerbations of chronic obstructive pulmonary disease. Clin Drug Investig.

[17] Aizzat O, Yap SW, Sopiah H, Madiha MM, Hazreen M, Shailah A, Wan JW, Nur SA, Srijit D, Musalmah M, Yasmin AM (2010). Modulation of oxidative stress by Chlorella vulgaris in streptozotocin (STZ) induced diabetic sprague-dawley rats. Adv Med Sci.

[18] Sano T, Tanaka Y (1987). Effect of dried, powdered Chlorella vulgaris on experimental atherosclerosis and alimentary hypercholesterolemia in cholesterol-fed rabbits. Artery.

[19] Sansawa H, Takahashi M, Tsuchikura S, Endo H (2006). Effect of chlorella and its fractions on blood pressure, cerebral stroke lesions, and life-span in stroke-prone spontaneously hypertensive rats. J Nutr Sci Vitaminol.

[20] Nakashima Y, Ohsawa I, Konishi F, Hasegawa T, Kumamoto S, Suzuki Y, Ohta S (2009). Preventive effects of chlorella on cognitive decline in age-dependent dementia model mice. Neurosci Lett.

[21] Kim YJ, Jeong S, Kwon S, Kim MK (2009). Effect of Chlorella vulgaris intake on antioxidative capacity in rats oxidatively stressed with dietary cadmium. Food Sci Biotechnol.

[22] Vijayavel K, Anbuselvam C, Balasubramanian MP (2007). Antioxidant effect of the marine algae Chlorella vulgaris against naphthalene-induced oxidative stress in the albino rats. Mol Cell Biochem.

